# Place of Colistin-Rifampicin Association in the Treatment of Multidrug-Resistant *Acinetobacter Baumannii* Meningitis: A Case Study

**DOI:** 10.1155/2016/8794696

**Published:** 2016-03-15

**Authors:** Dahraoui Souhail, Belefquih Bouchra, Badia Belarj, Rar Laila, Frikh Mohammed, Oumarou Mamane Nassirou, Ibrahimi Azeddine, Charki Haimeur, Abdelhay Lemnouer, Mostafa Elouennass

**Affiliations:** ^1^Equipe de Recherche Epidémiologie et Résistance Bactérienne, Service de Bactériologie Hôpital, Militaire d'instruction Mohammed V, 10100 Rabat, Morocco; ^2^Pôle Anesthésie Réanimation Hôpital, Militaire d'instruction Mohammed V, Faculté de Médecine et de Pharmacie, Université Mohammed V, BP 6203, Rabat, Morocco; ^3^Biotechnology Laboratory (MedBiotech), Rabat Medical & Pharmacy School, Mohammed V Rabat University, BP 6203, Rabat, Morocco

## Abstract

Treatment of* Acinetobacter baumannii* meningitis is an important challenge due to the accumulation of resistance of this bacteria and low meningeal diffusion of several antimicrobial requiring use of an antimicrobial effective combination to eradicate these species. We report a case of* Acinetobacter baumannii* multidrug-resistant nosocomial meningitis which was successfully treated with intravenous and intrathecal colistin associated with rifampicin.

## 1. Background

According to the World Health Organization (WHO) [1], antibiotic resistance is one of the three most important health problems. Treatment of* Acinetobacter baumannii (Ab)* meningitis is a real challenge, especially because of the bacteria ability to develop resistance and the antibiotics low delivery cross the blood-brain barrier. The WHO recorded* Acinetobacter baumannii* as a nosocomial pathogen of which antibiotic resistance is a threat to public health [2]. We report a case of postsurgical multidrug-resistant* Acinetobacter baumannii (MR-Ab)* meningitis which was successfully treated with an intravenous and an intrathecal colistin combined with rifampicin.

## 2. Case Presentation

A 42-year-old woman polytraumatized with cranial and thoracic impacts due to traffic accident was admitted to the surgical intensive care unit of the Mohammed Vth military teaching Hospital of Rabat.

**Figure 1:**
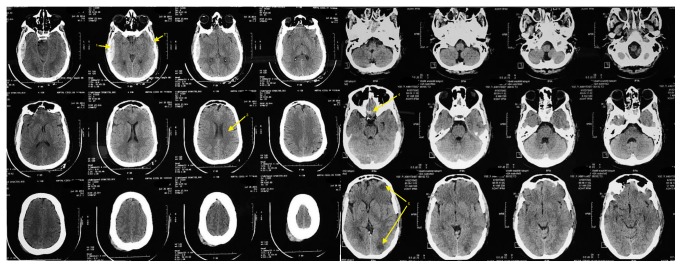
Computed tomography of patient at admission. (1) Extra-dural hematoma of 15 mm. (2) Pneumocephalus. (3) Cerebral edema. (4) Contusions of frontal brain lobe. (5) Extensive ischemia of the entire right hemisphere.

At admission, patient had a Glasgow Coma Score of 5, bilateral fractures of temporal bones, bilateral contusions of frontal brain lobes, pneumocephalus, pneumospin, and extensive ischemia of the entire right hemisphere and an extra-dural hematoma of 15 mm which was drained urgently (Figure 1).

Biological testing showed leukocytosis with 22.000 cells per microliter and a C-reactive protein at 66 milligrams per liter. The patient received a treatment of Ceftriaxone 2 g/day + gentamicin 160 mg/day for 7 days.

Eighth days after admission, the patient presented febrile peaks (39.5°C) and abundant purulent lung secretions with an evolutive radiological image. Her blood Leukocytes increased (33.000 cell per microliter) and the C-reactive protein was at 156 milligrams per liter. The patient did not have procalcitonin measurement due to the cost of the testing; only C-reactive protein was used for inflammation monitoring.

Blood cultures, and bacteriological examination of the femoral catheter, the tracheal aspirate (TA), and cerebrospinal fluid (CSF) (lumbar puncture was done on lateral decubitus position) were performed.

Cytobacteriological examination of CSF revealed a leukocyte count of 700 per cubic millimeter with 73% of neutrophils and 27% of lymphocytes. Red blood cells count was 11200 per cubic millimeter. The CSF glucose level was at 0.6 grams per liter and protein concentration was at 2.30 gram per liter.

Gram staining of CSF found rare Gram negative coccobacilli. The CSF culture found abundant sprout of MR-Ab producing a carbapenemase. The MR-Ab CSF strain was susceptible to colistin (MIC colistin = 1.5 *μ*g/mL), rifampicin (MIC rifampicin = 4 *μ*g/mL), netilmicin, amikacin, and ampicillin + sulbactam while it was resistant to cotrimoxazole, ticarcillin, ticarcillin + clavulanic acid, piperacillin, piperacillin + tazobactam, cefepime, ceftazidime, imipenem, gentamicin (10 *μ*g), tobramycin, ciprofloxacin, and chloramphenicol.

The TA culture isolated 107 CFU/mL MR-Ab strain susceptible only to colistin [minimum inhibitory concentration (MIC) of colistin = 1.5 *μ*g/mL] and rifampicin. 106 CFU/mL strain of wild-type phenotype* Pseudomonas aeruginosa* was isolated on also TA cultures. A similar strain of MR-Ab was isolated on a catheter crop of the central femoral lane and on the blood culture (Table 1).

The diagnosis of MR-Ab nosocomial meningitis, and bacteremia, and respiratory coinfection with MR-Ab and* Pseudomonas aeruginosa* was established.

Treatment was switched on day 10 to 125000 UI colistin intrathecal injections for two days, in addition to intravenous colistin (4 MIU every 8 hours) and intravenous imipenem (1 g every 8 hours). 72 hours later and as there was no clinical nor biological improvement, rifampicin was added (600 mg intravenous shot two times a day). Intravenous colistin and rifampicin were given for 21 days; imipenem was given for 14 days.

Apyrexia was obtained after nine days, blood leukocytes was improved (10200/cubic millimeter), and C reactive protein was at 26 milligrams per liter. Chest X-ray image was normalized and TA cultures after treatment did not isolate pathogens.

At discharge, the computed tomography control scan showed a slight decrease in right-sided ischemia injuries. The clinical examination found residual left-sided hemiplegia, hypertensive peaks, and a pressure ulcer at the buttock. No other neurological deficits left.

## 3. Conclusions

Ab nosocomial meningitis is rare and accounts only for 4% of nosocomial meningitis [3]. The mortality rate of nosocomial meningitis is estimated at 15% [4] and increases to 40% if the causing agent is Ab [5]. Moreover, this rate may be up to 70% in developing countries [6]. For our patient, the contamination could have occurred directly from a skin colonized by Ab which is a hospital environment bacteria or through the blood circulation from the lung infection site. Indeed, even if it is not clearly defined as a risk factor, Ab colonization seems to increase the risk of developing nosocomial meningitis [7–9]. However, given the similarities of the isolates in the CSF and in the blood and catheter culture, infection origin seems to be bacteremia.

Colistin is considered an efficient treatment option for MR-Ab infections for patients in intensive care units [10]. The most common toxicity of this treatment is nephrotoxicity [11]. However, recent studies have shown that this nephrotoxicity seems to be less important [12] as Garnacho-Montero et al. [13] noticed in a prospective study conducted on 1205 patients demonstrating no significant differences in appearance of a kidney failure between colistin and imipenem. In another prospective study carried out by Falagas et al. [14], the intravenous injection of colistin did not lead to any nephrotoxicity for a high proportion of patients. However, the same study clearly showed that there was a correlation between the cumulative dose of colistin and an increase in creatinine serum concentration. Our patient did not have any side effect after 7 days of withdrawal of the colistin and the serum creatinine urea levels were, respectively, 9 mg/L and 0.15 mg/L.

On the other hand, colistin diffusion to the CSF is a real issue for nosocomial meningitis management. Jiménez-Mejías et al. show that only 25% of administered colistin diffuses to the CSF [15]. In order to achieve the effective concentration, the use of intrathecal colistin on MR-Ab meningitis is highly recommended [16–18]. There are few data defining the exact doses of intrathecal colistin in meningitis but the Infectious Diseases Society of America recommends 125 000 UI (10 mg) of intrathecal colistin [19]. In fact, clinical and biological data of our patient only improved after intrathecal colistin shot associated with rifampicin.

This antibiotic has proved to be an efficient treatment of meningitis caused by imipenem resistant Ab strains due to its excellent diffusion in CSF. Moreover, rifampicin and colistin have synergistic effects on the treatment of central nervous system infections due to Ab [20]. However, the high risk of resistant mutants' selection after only 24 hours reduces rifampicin use in a monotherapy [21]. In that regard, Pachón-Ibáñez et al., in a mouse experimental pneumonia model, recommends the association of rifampicin to imipenem if imipenem MIC is not higher than 32 mg/L [21]. For our patient, an early combination of imipenem and rifampicin could have prevented Ab resistance development.

The great ability of Ab to develop resistance is a worrying issue. However, the combined use of intravenous and intrathecal colistin with rifampicin and imipenem appears to be an efficient treatment option for Ab-MR meningitis and further clinical studies should be done to confirm the effectiveness of this combination.

## Figures and Tables

**Table 1:** Comparison of antibiotics susceptibility of *Ab* strains isolated from the different patient samples.

Antibiotics	CSF	Blood culture	Femoral catheter	BA
Colistin	S (MIC = 1.5 *µ*g/mL)	S	S	S (MIC = 1.5 *µ*g/mL)
Rifampicin	S (MIC = 4 *µ*g/mL)	S	S	S
Netilmicin	S	S	S	R
Amikacin	S	S	S	R
Ampicillin + sulbactam	S	S	S	R
Cotrimoxazole	R	R	R	R
Ticarcillin	R	R	R	R
Ticarcillin + clavulanic acid	R	R	R	R
Piperacillin	R	R	R	R
Piperacillin + tazobactam	R	R	R	R
Cefepime	R	R	R	R
Ceftazidime	R	R	R	R
Imipenem	R	R	R	R
Gentamicin, 10 *µ*g	R	R	R	R
Tobramycin	R	R	R	R
Ciprofloxacin	R	R	R	R
Chloramphenicol	R	R	R	R

R: resistant; S: susceptible.

## Abbreviations

Ab: * Acinetobacter baumannii*
MR-Ab: Multidrug-resistant* Acinetobacter baumannii*
MIC: Minimum inhibitory concentration
CSF: Cerebrospinal fluid
MTB-MDR: * Mycobacterium tuberculosis* multidrug-resistant
WHO: World Health Organization.
